# Handover patterns: an observational study of critical care physicians

**DOI:** 10.1186/1472-6963-12-11

**Published:** 2012-01-10

**Authors:** Roy Ilan, Curtis D LeBaron, Marlys K Christianson, Daren K Heyland, Andrew Day, Michael D Cohen

**Affiliations:** 1 Department of Medicine and Critical Care Program, Queen's University, Kingston General Hospital, Etherington Hall, Room 1005, 94 Stuart Street, Kingston, ON, Canada, K7L 3N6; 2 Department of Organizational Leadership & Strategy, Marriott School of Management, Tanner Building 790, Brigham Young University, Provo, Utah 84602, USA; 3 Rotman School of Management, 105 St. George Street, Toronto, ON, Canada, M5S 3E6; 4 Clinical Research Centre, Kingston General Hospital, Kingston, ON, Canada, K7L 3N6; 5 School of Information, 312 West Hall, School of Public Policy, 407 Lorch Hall, University of Michigan, Ann Arbor, Michigan 48109-1092, USA

**Keywords:** Adverse effects, Communication, Safety, Standardization, Video Recording

## Abstract

**Background:**

Handover (or 'handoff') is the exchange of information between health professionals that accompanies the transfer of patient care. This process can result in adverse events. Handover 'best practices', with emphasis on standardization, have been widely promoted. However, these recommendations are based mostly on expert opinion and research on medical trainees. By examining handover communication of experienced physicians, we aim to inform future research, education and quality improvement. Thus, our objective is to describe handover communication patterns used by attending critical care physicians in an academic centre and to compare them with currently popular, standardized schemes for handover communication.

**Methods:**

Prospective, observational study using video recording in an academic intensive care unit in Ontario, Canada. Forty individual patient handovers were randomly selected out of 10 end-of-week handover sessions of attending physicians. Two coders independently reviewed handover transcripts documenting elements of three communication schemes: SBAR (Situation, Background, Assessment, Recommendations); SOAP (Subjective, Objective, Assessment, Plan); and a standard medical admission note. Frequency and extent of questions asked by incoming physicians were measured as well. Analysis consisted of descriptive statistics.

**Results:**

Mean (± standard deviation) duration of patient-specific handovers was 2 min 58 sec (± 57 sec). The majority of handovers' content consisted of recent and current patient status. The remainder included physicians' interpretations and advice. Questions posed by the incoming physicians accounted for 5.8% (± 3.9%) of the handovers' content. Elements of all three standardized communication schemes appeared repeatedly throughout the handover dialogs with no consistent pattern. For example, blocks of SOAP's *Assessment *appeared 5.2 (± 3.0) times in patient handovers; they followed *Objective *blocks in only 45.9% of the opportunities and preceded *Plan *in just 21.8%. Certain communication elements were occasionally absent. For example, SBAR's *Recommendation *and admission note information about the patient's *Past Medical History *were absent from 22 (55.0%) and 20 (50.0%), respectively, of patient handovers.

**Conclusions:**

Clinical handover practice of faculty-level critical care physicians did not conform to any of the three predefined structuring schemes. Further research is needed to examine whether alternative approaches to handover communication can be identified and to identify features of high-quality handover communication.

## Background

Handover, or an equivalent term 'handoff', is the exchange between health professionals of information about a patient accompanying either a transfer of control over, or of responsibility for, the patient [1]. Miscommunication during transfer of care for hospitalized patients is common and can result in adverse events [2-7].

Guidelines and recommendations for handover practices have been proposed by patient safety organizations around the world [8-13]. In particular, the use of standardized approaches during handovers, including mnemonics that establish topics and their sequence, has been promoted and adopted by accreditation committees [14,15].

However, most suggested recommendations are based on expert opinions and have not been shown to improve communication effectiveness or patient safety. Furthermore, the available knowledge on physicians' handovers is based predominantly on studies of medical trainees [11,16,17]. In a recent literature review, Reisenberg and colleagues identified only one study that did not involve residents [17]. Our study seeks to uncover the existing practices of experienced physicians (attending physicians rather than residents) with an aim to compare their communication approaches to proposed standards. Our research acknowledges the inherent wisdom of everyday practice -- that is, we recognize that people and professionals have an abundant capacity to figure out what works for them in a particular situation [18-20]. The intensive care unit (ICU), replete with potent and complex interventions, is a good place to study naturally occurring handovers. Accordingly, we conducted an observational study to characterize and assess handover patterns among attending critical care physicians. In the present study we consider a specific issue of direct policy significance: Since many calls have been issued for standardization of handovers, we examine the extent to which handover practices of our recorded attending physicians do in fact conform to currently popular structuring schemes that might serve as handover standards. Thus, our research objective is to describe handover communication patterns used by the participating physicians and to compare them to widely promoted, standardized handover communication patterns. Our findings should inform future research, educational and quality improvement activities in the area of handover.

## Methods

### Study Design, Setting and Participants

We conducted a prospective observational study of handover sessions between critical care attending physicians at Kingston General Hospital, a 456-bed tertiary centre in Ontario, Canada, during November 2008 to July 2009. The ICU is an academic 21-bed unit providing care for medical, surgical, trauma and cardiovascular surgery patients. In addition to attending physicians, medical teams include residents at different training levels and critical care fellows. Two attending physicians are assigned to the ICU for one-week periods, which start on Fridays. They split patient care and on-call responsibilities during weekdays, while a single physician is in charge during weekends. Attending physician handovers usually take place on Thursdays, typically in the form of oral communication, either in person or, less frequently, over the phone.

We created a data bank by videotaping naturally-occurring handovers between critical care attending physicians, which usually took place in a conference room within the ICU. In this study, we analyzed a convenience sample of these data consisting of 21 handover sessions. Our study is based on transcriptions of randomly sampled portions of those sessions. Throughout our data collection, we were careful not to alter the behaviour and setting that we were observing. Two of the investigators (RI, DH) participated as subjects in the study by continuing in their role as attending physicians and by conducting handovers as usual. However, all participants were unaware of the research question, as well as the specific nature of the analyses that would be conducted. For each participating physician, one handover session (as an outgoing physician) was randomly selected. From each sampled session, the handovers of four patients were randomly selected using a random number generator. Selected handovers were transcribed; identifiers (e.g., names) were removed from the recordings and transcripts.

This study was approved by the Research Ethics Board of the Queen's University School of Medicine. Signed informed consent was obtained from all participating physicians.

### Measures

Handover activity is conducted in a complex organizational context and can be analyzed at many different levels. Since the commonly promoted handover standards are defined at the patient handover level, our analysis is based on a sampling of patients from within a sample of sessions.

The content of handovers was examined according to three well known standardized communication schemes, including SBAR (*Situation, Background, Assessment, Recommendations*), SOAP (*Subjective, Objective, Assessment, Plan*), and the medical admission note (MAN). The proportion, absence and recurrence of various elements of the selected standardized schemes were observed and documented.

SBAR is a mnemonic reminding users to present the patient's *Situation *and *Background*, followed by the clinician's *Assessment *and *Recommendations*. Although SBAR was designed for nurse briefings of physicians in an obstetrics unit that were not handovers [21], the tool has been presumed to be generally useful for the handover situation; it has been widely promoted as a handover standardized procedure [22-26]. In a literature review of 46 papers presenting handover mnemonics, Riesenberg et al found that 69.6% mentioned SBAR [16]. SOAP is another mnemonic widely espoused by physicians in accordance with the problem-oriented medical record. It reminds physicians to consider and record the patient's *Subjective *symptoms and complaints, and the *Objective *physical findings and test results, as well as the physician's *Assessment *and management *Plan *[27]. SOAP has been suggested as a handover standard [28]. The medical admission note (MAN) regularly used in our hospital (and ICU) includes the following headings: patient identification; chief complaint or reason for ICU admission; history of present illness; past medical history, review of systems and family history; regular medications, habits and allergies; social history; findings on physical examination and test results; and problem formulation and management plan. The MAN, although not specifically designed for handover communication, is likely the most detailed type of medical record and therefore allows for good discrimination between different communication contents.

Questions are generally assumed to have significant potential for improving the quality of handover conversations. For example, questions during handovers may lead to new clinical insights or to error detection and correction [29,30]. Some have tried to extend and improve the SBAR scheme by adding a "Q" (for "questions") to the mnemonic [31]. Thus, we coded for the frequency and extent of questions asked by the incoming physicians, treating questions as a distinct element, not overlapping with any of the other standard elements.

There have been many other proposed handover standards, but those we examine here are by far the most common and most likely to match the actual behaviour of our expert participants [1].

**Figure 1:**
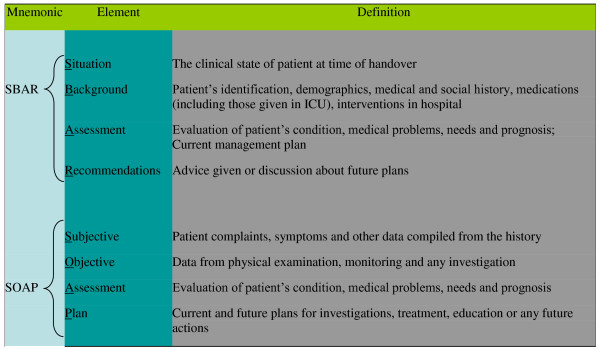
**Definitions of SBAR and SOAP**.

**Figure 2:**
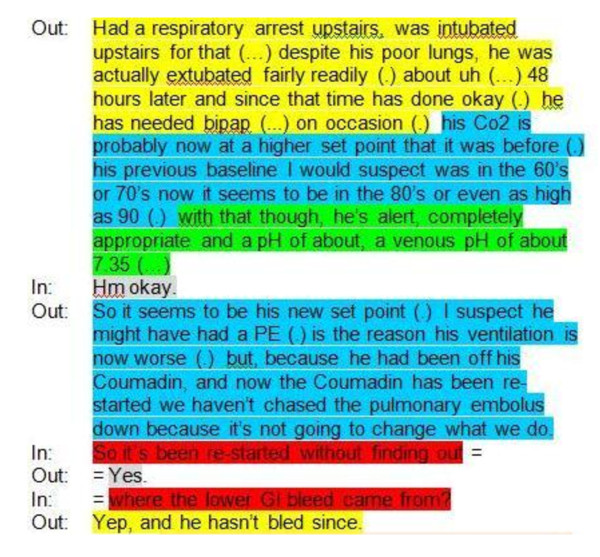
**Example of a coded transcript according to SBAR and Questions**. SBAR, Situation, Background, Assessment, Recommendation; Out (outgoing), handing over physician; In (incoming), physician receiving information. Color codes: Green = Situation; Yellow = Background; Light blue = Assessment; Purple = Recommendation; Red = Question; Grey = Other.

The elements composing the various mnemonics were predefined by the research team in accordance with previous publications [24,27] (Figure 1). Transcripts of the selected handovers were then independently color-coded for each of the three communication schemes (Figure 2) by two out of three coders, including a 3^rd ^year medical student, a social worker and a critical care fellow. The coders were trained by the primary investigator (RI) prior to coding the transcripts. The training included in-person instruction regarding the various mnemonics and the definitions of the specific elements, and a supervised coding of one handover transcript. Throughout the coding process, coders' questions and uncertainties were brought to the primary investigator for a final adjudication. There was no attempt to resolve disagreement among the coders. Total duration of handover sessions was measured from the video recording. The quantity of each communication element was measured by word count from the coded transcripts. For the purpose of determining the total word count, all non-alphanumeric content (e.g. punctuation marks) was excluded. Proportions of different elements of the communication schemes were calculated using the number of words or blocks (i.e. consecutive words coded as the same element) relevant to each section over the total number of words or blocks, respectively, in the transcript. Recurrence of specific elements of the various patterns in each patient's handover was defined by counting discontinuous blocks of each element. Demographic data of participating physicians and the setting of each handover were recorded.

### Analysis

Statistical analysis was generated by SAS 9.1.2 (SAS Institute, Cary, North Carolina). Descriptive statistics were used to characterize participant demographic and clinical data, handover characteristics, and proportion and recurrence rates of various elements of the communication schemes. The internal consistency among coders was assessed using the kappa statistic [32]. To check for potential differences in approach among physicians, one-way ANOVA was used to examine the variance in handover durations among physicians.

## Results

### Study Sample

During the study's 34 weeks, a total of 21 handover sessions were recorded, including 15 sessions with a single outgoing and a single incoming physician; 2 sessions with two outgoing and a single incoming; and 4 sessions with two outgoing and two incoming physicians. Ten out of 11 critical care attending physicians agreed to participate in the study and be videotaped. They conducted a mean (± standard deviation) of 2.3 (± 1.3) sessions in the outgoing role and were in the incoming role in 2.1 (± 1.4) sessions each. Participating physicians' age, sex, primary specialty and years of experience as an attending physician are shown in Table 1.

**Table 1:** Characteristics of participating critical care attending physicians; n = 10

Age, mean (SD)	44.6 (8.3)
Sex, male (%)	9 (90.0)
Primary Specialties, n (%)	
Respirology	3 (30.0)
Emergency Medicine	2 (20.0)
General Surgery	2 (20.0)
Internal Medicine	2 (20.0)
Anesthesiology	1 (10.0)
Years as Attending Physician, median (interquartile range)	9.0 (2.5-15.0)

SD, standard deviation

### General Characteristics

Nine of the 10 randomly selected handover sessions were performed face to face in a conference room at the ICU; 1 session was performed over the phone. Handover sessions included a mean of 9.5 (± 2.2) patients. Mean durations of the full sessions and of patient-specific handovers were 30 min 3 sec (± 11 min 7 sec) and 2 min 58 sec (± 57 sec), respectively. Handover durations (range, 9 sec-7 min 19 sec) varied among physicians and 29% of the variance is explained by the physicians (*p *< 0.001). There was no significant correlation between physicians' years of experience and these handover durations.

Neither the hospital nor the ICU has a policy about how handovers should be conducted. According to their individual preference or practice, outgoing physicians used printed materials, including patient lists and personal notes, while incoming physicians often took notes.

### Handover Content

Table 2 shows the proportion, absence and recurrence rates for SBAR, SOAP and MAN elements, as well as questions in the handovers. Rates according to two coders and averages of both are presented. Overall level of agreement between the two coders was good for SBAR (*Kappa*, 0.57), poor for SOAP (*Kappa*, 0.38) and good for elements of MAN (*Kappa*, 0.69). In the following lines average rates are reported.

**Table 2:** Proportion, absence and appearance rates of SBAR, SOAP and MAN elements in patients' handovers

		Proportion, % (± SD)			Absence, n (%)			Appearances per Patient, n (± SD)		
		Coder 1	Coder 2	Average	Coder 1	Coder 2	Average	Coder 1	Coder 2	Average
	S	5.2 (5.6)	26.3 (14.4)	**15.8 (8.4)**	8 (20.0)	1 (2.5)	**1 (2.5)**	2.1 (1.8)	4.5 (2.8)	**3.3 (2.1)**
**SBAR**	B	67.6 (11.2)	45.3 (15.1)	**56.4 (11.6)**	0 (0.0)	0 (0.0)	**0 (0.0)**	4.9 (2.6)	3.5 (2.2)	**4.2 (2.2)**
	A	18.8 (8.9)	20.3 (10.1)	**19.5 (9.0)**	1 (2.5)	2 (5.0)	**1 (2.5)**	4.3 (2.6)	4.5 (2.6)	**4.4 (2.2)**
	R	2.6 (3.9)	2.3 (3.8)	**2.4 (3.7)**	23 (57.5)	25 (62.5)	**22 (55.0)**	0.5 (0.7)	0.4 (0.6)	**0.5 (0.6)**

	S	26.2 (11.1)	54.4 (17.3)	**40.5 (11.4)**	1 (2.5)	1 (2.5)	**1 (2.5)**	2.9 (1.6)	7.4 (4.1)	**5.1 (2.5)**
**SOAP**	O	18.2 (9.7)	13.6 (9.3)	**15.9 (7.2)**	2 (5.0)	1 (2.5)	**0 (0.0)**	4.1 (2.7)	4.7 (3.1)	**4.4 (2.7)**
	A	31.8 (14.1)	20.6 (9.2)	**26.0 (8.2)**	0 (0.0)	1 (2.5)	**0 (0.0)**	5.0 (3.3)	5.3 (3.5)	**5.2 (3.0)**
	P	17.7 (11.3)	5.6 (6.5)	**11.6 (8.0)**	3 (7.5)	17 (42.5)	**3 (7.5)**	2.8 (2.0)	1.2 (1.5)	**2.0 (1.4)**

	ID	1.8 (0.9)	2.1 (0.7)	**2.0 (0.7)**	8 (20.0)	6 (15.0)	**6 (15.0)**	0.9 (0.5)	1.2 (0.7)	**1.0 (0.6)**
	CC	0.6 (0.5)	0.3 (0.4)	**0.5 (0.4)**	33 (82.5)	36(90.0)	**35 (87.5)**	0.2 (0.4)	0.1 (0.3)	**0.1 (0.3)**
	HPI	24.6 (6.9)	33.5 (4.0)	**29.1 (4.3)**	1 (2.5)	1 (2.5)	**1 (2.5)**	3.4 (2.1)	9.2 (4.8)	**6.3 (4.7)**
	PMH	5.7 (3.7)	2.0 (1.7)	**3.8 (2.4)**	14 (35.0)	25 (62.5)	**20 (50.0)**	1.3 (1.4)	0.8 (1.2)	**1.0 (1.4)**
**MAN**	MHA	2.2 (1.6)	0.5 (0.7)	**1.3 (1.0)**	20 (50.0)	33 (82.5)	**27 (67.5)**	0.9 (1.3)	0.3 (0.8)	**0.6 (1.1)**
	Social	2.0 (2.0)	3.1 (3.5)	**2.5 (2.5)**	28 (70.0)	21 (52.5)	**25 (62.5)**	0.5 (0.8)	0.9 (1.2)	**0.7 (1.1)**
	DOC	3.7 (4.5)	0.8 (0.8)	**2.2 (2.5)**	29 (72.5)	33 (82.5)	**31 (77.5)**	0.5 (1.0)	0.3 (0.6)	**0.4 (0.8)**
	PE	3.5 (1.7)	4.5 (2.8)	**4.0 (1.9)**	16 (40.0)	11 (27.5)	**14 (35.0)**	1.0 (1.1)	1.9 (2.1)	**1.5 (1.7)**
	Tests	12.6 (6.6)	10.5 (4.9)	**11.6 (5.5)**	8 (20.0)	6 (15.0)	**7 (17.5)**	2.9 (2.6)	3.3 (2.8)	**3.1 (2.7)**
	A+P	43.2 (9.4)	42.7 (6.7)	**42.9 (7.3)**	0 (0.0)	0 (0.0)	**0 (0.0)**	7.4 (4.1)	11.4 (6.5)	**9.4 (5.8)**

**Questions**		5.7 (3.9)	5.9 (3.9)	**5.8 (3.9)**	2 (5.0)	2 (5.0)	**2 (5.0)**	3.9 (3.6)	3.9 (3.3)	**3.9 (3.3)**

SBAR, Situation, Background, Assessment, Plan; SOAP, Subjective, Objective, Assessment, Plan; MAN, medical admission note; SD, standard deviation; ID, identification; CC, chief complaint; HPI, history of present illness; PMH, past medical history; MHA, medications, habits and allergies; DOC, directives of care; PE, physical examination; A+P, assessment and plan.

For SBAR, the major part of handover content, 56.4% (± 11.6%), included *Background *elements. Much of the discordance among coders resulted from disagreement on *Situation *and *Background *(*Kappa*, 0.28). The *Recommendation *element was completely absent from 22 (55.0%) of the patient handovers.

For SOAP, *Subjective *elements comprised 40.5% (± 11.4%) and *Assessment *26.0% (± 8.2%) of handover content. The *Plan *element was absent from 3 (7.5%) of the handovers.

For MAN, expressions of *Assessment *and descriptions of *History of Present Illness *comprised 42.9% (± 7.3%) and 29.1% (± 4.3%), respectively, of handover content. With the exception of *Assessment*, all MAN elements were occasionally absent from handovers (Table 2).

Questions were asked by the incoming physicians 3.9 (± 3.3) times during an average handover and accounted for 5.8% (± 3.9%) of the total handover content. Questions were not asked at all in handovers of just 2 (5.0%) patients.

The proportions, as well as rates of absence and repetitions of all SBAR, SOAP and MAN elements, were similar when excluding data of the two participating investigators (RI, DH) from the analysis.

Table 3 shows the proportions of elements opening ("First") and closing ("Last") the handovers, as well as the arrangement of SBAR, SOAP and MAN elements throughout the handovers, averaged over the two coders.

**Table 3:** Proportion of elements opening ("First") and closing ("Last") the handovers and succession of SBAR, SOAP and MAN elements*, ^@^

SBAR													
	
				Followed by									
					
		First	Last	S			B		A		R		
	S	13.8	21.3				38.9		57.4		3.7		
SBAR	B	77.5	26.3	36.3					59.6		4.1		
	A	8.8	38.8	39.9			54.7				5.3		
	R	0.0	13.8	28.6			25.0		46.4				
SOAP													
	
				Followed by									
					
		First	Last	S			O		A		P		
	S	66.3	21.3				44.9		45.4		9.7		
SOAP	O	20.0	11.3	43.0					45.9		11.1		
	A	10.0	41.3	45.0			33.2				21.8		
	P	3.8	26.3	26.2			23.4		50.4				
MAN													
				Followed by									
				
		First	Last	ID	CC	HPI	PMH	MHA	Social	DOC	PE	Tests	A+P
	ID	62.5	2.5		4.1	51.4	32.4	2.7	0.0	0.0	0.0	1.4	8.1
	CC	0.0	0.0	0.0		45.5	0.0	9.1	0.0	0.0	0.0	45.5	0.0
	HPI	7.5	2.5	2.5	2.2		3.7	2.5	2.2	1.9	8.0	23.5	53.6
**MAN**	PMH	7.5	0.0	3.2	0.0	58.1		9.7	3.2	0.0	3.2	9.7	12.9
	MHA	0.0	0.0	0.0	0.0	20.9	9.3		7.0	0.0	4.7	11.6	46.5
	Social	0.0	3.8	0.0	2.4	14.6	0.0	7.3		4.9	2.4	0.0	68.3
	DOC	2.5	2.5	0.0	0.0	19.0	0.0	0.0	0.0		9.5	9.5	61.9
	PE	0.0	5.0	0.0	0.0	30.3	2.0	1.0	1.0	0.0		16.2	49.5
	Tests	0.0	3.8	0.0	0.0	25.1	1.9	4.2	0.9	0.0	11.6		56.3
	A+P	20.0	80.0	4.3	0.0	37.0	2.7	3.5	7.8	3.5	12.2	28.9	

* Averaged over the two coders; all numbers are in percentages. SBAR, Situation, Background, Assessment, Plan; SOAP, Subjective, Objective, Assessment, Plan; MAN, medical admission note; ID, identification; CC, chief complaint; HPI, history of present illness; PMH, past medical history; MHA, medications, habits and allergies; Social, social history; DOC, directives of care; PE, physical examination; A+P, assessment and plan.

^@^An example for data interpretation: For SBAR, *Background *elements opened 77.5% and closed 26.3% of the handovers. Whenever *Background *elements appeared and were not last, they were followed by *Situation, Assessment*, and *Recommendation *elements in 36.3%, 59.6%, and 4.1% of the opportunities, respectively.

### Opening and Closing

For SBAR, 77.5% of the handovers were started with *Background *and only 13.8% with *Situation *information. Only 13.8% of handovers were concluded with a *Recommendation*. For SOAP, 66.3% of the handovers were started with *Subjective *information and only 26.3% ended with a *Plan*. For MAN, the majority (62.5%) of handovers were started with identifying information, although 20.0% were started with *Assessment and Plan*, which also closed most (80.0%) handovers.

### Ordering of Elements

For SBAR and SOAP, arrangements of the various communication elements were similar: The first 3 elements (SBAR's *Situation*, *Background *and *Assessment *and SOAP's *Subjective*, *Objective *and *Assessment*) followed each other almost randomly. For example, SBAR's *Situation *was followed by *Background *information in 38.9% and by *Assessment *in 57.4% of the handovers. The last elements (SBAR's *Recommendation *and SOAP's *Plan*) followed any of the other elements to a lesser extent. For MAN, most elements were followed by *Assessment and Plan*. Exceptions include *Identification*, which was usually followed by *History of Present Illness *(51.4%) or *Past Medical History *(32.4%); *Chief Complaint*, which was absent in 87.5% of the handovers; and *Past Medical History*, which was usually followed by *History of Present Illness *(58.1%). Only a single element, *Tests*, was followed by the "appropriate" element (in this case *Assessment and Plan*, which, in fact, followed most other elements) in most handovers (56.3%).

**Figure 3:**
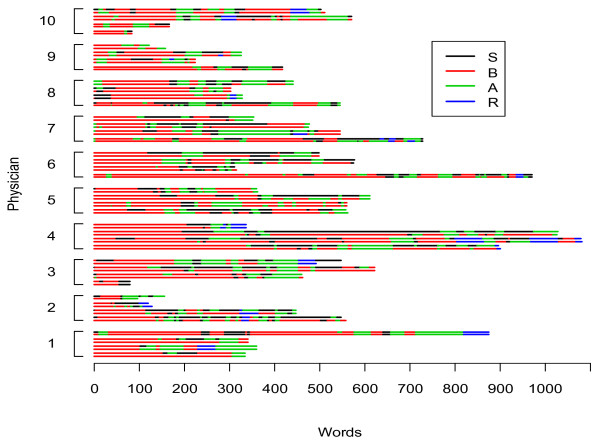
**Occurrence of SBAR elements**. Two coders shown for four sampled patient handovers for each of 10 handing-over physicians.* S, Situation; B, Background; A, Assessment; R, Recommendation. * Data are for the conversation in which physician (i) was handing over, but the data include what was said in the conversation by the incoming physician as well.

**Figure 4:**
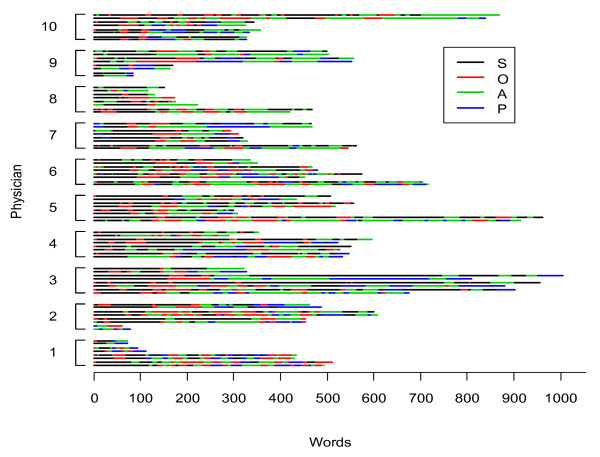
**Occurrence of SOAP elements**. Two coders shown for four sampled patient handovers for each of 10 physicians.* S, Subjective; O, Objective; A, Assessment; P, Plan. * Data are for the conversation in which physician (i) was handing over, but the data include what was said in the conversation by the incoming physician as well.

Figure 3 and Figure 4 show the occurrence of SBAR and SOAP elements, respectively, in the 40 handovers that we analyzed. In addition to the dominance of *Background *and *Subjective *elements (for SBAR and SOAP, respectively), these figures illustrate the mixed arrangement and multiple occurrences of all the mnemonic elements.

**Figure 5:**
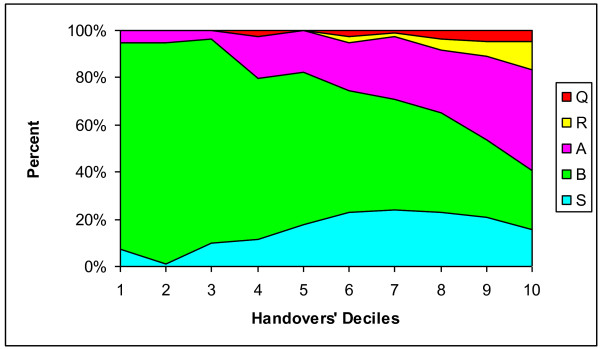
**Proportion of SBAR+Q elements in each ten percent block of standardized handover length, averaged over 40 handovers and 2 coders**. S, Situation; B, Background; A, Assessment; R, Recommendation; Q, Question.

**Figure 6:**
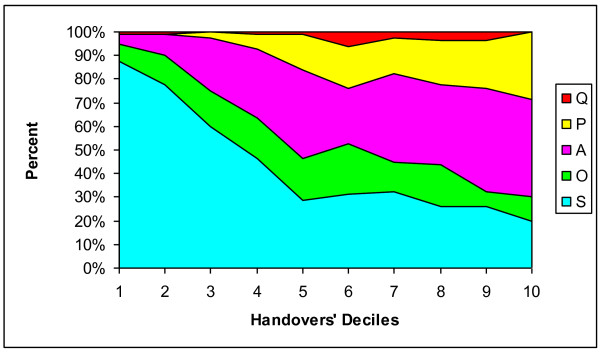
**Proportion of SOAP+Q elements in each ten percent block of standardized handover length, averaged over 40 handovers and 2 coders**. S, Subjective; O, Objective; A, Assessment; P, Plan; Q, Question.

Figure 5 and Figure 6 represent the proportion of SBAR, SOAP and Question elements in each ten percent block of handover length averaged over 40 handovers and 2 coders. Figure 5 shows that *Background *information comprises about 90% of the content in the first 20-30 percent of an average handover, and then gradually decreases throughout the handover. *Situation *content is present in a fairly constant level whereas expressions of *Assessment *gradually increase throughout the handovers. *Recommendations *first appear at the second half of an average handover and then gradually increase. Figure 6 shows that *Subjective *aspects of the patient are the vast majority of material in the first 10 percent of an average handover, but fall to about 30 percent of the content by the middle of the handover and, on average, remain at about that level for the rest of the discussion. *Objective *elements, in contrast, are present at a moderate but constant level throughout the 40 handovers.

## Discussion

During end-of-week handovers in the ICU, the observed attending physicians spent about 3 minutes discussing each patient. Elements of the three communication schemes (SBAR, SOAP, MAN), often completely absent, were distributed throughout the handovers.

Although SBAR, originally developed to achieve effective briefing during critical situations [21,24], is relatively new to the medical community, SOAP and MAN have been commonly promoted and could potentially be the physicians' default for the purpose of handover communication. Clearly, these physicians were taking a communication approach that is not standardized around a simple ordered list of topics. Apparently, these widely promoted schemes do not fit the clinical work required during handover.

Information about the patient's condition and history (*Situation *and *Background *elements in SBAR; *Subjective *and *Objective *in SOAP; and *History of Present Illness, Test Results *and *Past Medical History *elements in the MAN) comprised about half of the handovers' contents. Considering a usual week-long trajectory of critically ill patients in the ICU, the high proportion of "background" information in the handovers is not surprising. Interpretation of the patient's condition (*Assessment *in SBAR, SOAP and MAN) comprised about one third of the discussion.

Questions were asked commonly by incoming physicians: about 4 questions during each handover; questions not asked at all in only 2 out of the 40 handovers. Although the value of questions is often assumed to lie in verifying or completing the transmission of facts about the patient, questions may play a considerably larger role in the process of two physicians jointly constructing their picture of the patient. Questions can invite or solicit more information; initiate repair or correction of mistakes; seek confirmation of hearing and/or understanding; slow the conversation down for the purpose of note-taking; propose an alternative organization of information; display expertise or assert competence (someone knows enough to ask the question); provide a polite way of suggesting an alternative diagnosis or treatment possibility; and so forth. Through questions and other responses, incoming physicians become the co-authors of reports by outgoing physicians. Handovers are an interactive accomplishment, not a monologic and unilateral transfer of information. Furthermore, what constitutes a question is not always clear: human speech does not come with explicit question marks, so the occurrence of a question may be ambiguous, and that ambiguity may serve the micro-political ends of the participants, allowing for such things as diplomatic corrections or soft directions. Further research is needed to refine and analyze the types and functions of questions in handovers.

In our recorded handovers, different elements of the various communication schemes were continuously interwoven throughout the exchanges. This may suggest that the physicians chose to deliver their message by portraying a sensemaking, relevant story rather than spelling out the entirety of the available information about their patients [29,33]. An account of events leading to the patient's current condition is likely to include multiple segments of various types of information, as suited to the individual patient's medical problems. Utterances of medical history, test results and their interpretation, social history, directives of care, etc. may need to be carefully deployed-placed in narrative context, omitted in some cases, repeated in others-to achieve the supposed goal of a handover: an understanding of the patient's condition and needs, by the incoming physician, in a way that is memorable and can usefully inform the subsequent actions [3,34]. Attempting to rigidly apply any of the studied standardized approaches for delivering a handover might have failed to achieve this goal.

One of the most striking findings in our study relates to elements that were absent in handovers. In particular, *Recommendation *content was absent in about 60% of patient handovers, in the judgement of both coders. Lack of recommendation has also been observed in nurses practicing SBAR [35]. There are several possible explanations for this finding. First, it could be that the outgoing physicians have inadvertently omitted recommendations (i.e. error of omission) [36,37]. Second, because advice-giving and correcting may undermine the recipient's status [38], it may be that outgoing physicians did not offer recommendations out of respect for the incoming physicians' professional status [39,40]. Third, it may be that the outgoing physicians focused on conveying their sense of the patient's trajectory of care and what should be done next (recommendations) in narrative or story form, rather than a detailed list of what is wrong with the patient and what to do next [33,41,42]. Fourth, it may be that outgoing physicians sought to draw attention to potential problems of the patient even if they didn't have a clear sense of how to resolve those problems. Regardless of the reasons, a complete lack of recommendations or mention of management plans might lead to misperception by the incoming physician, and possibly to undesirable outcomes for patients and the organization. Further research is warranted into the reasons for and consequences of lack of peer-to-peer dialogue on treatment recommendations and into strategies that might improve physicians' discussion of such content.

For various reasons the quality of these recorded handovers cannot be evaluated at present. First, due to the complexity of the communication, and to the absence of the appropriate research, a "gold standard" for handover communication does not exist. Second, our small sample does not allow for a meaningful evaluation of clinical outcomes such as physician satisfaction, errors, indicators of inefficiency and adverse events. Further research is required to identify quality indicators for handovers. However, we believe that the observed communication of the studied physicians does indicate important features of expertise. It is unlikely that content elements were interspersed throughout the handovers simply because these attending physicians-with an average of 9 years of experience-lacked the ability to organize their thoughts and put together a coherent description of their patients. In fact, within about 3 minutes they summarized several days in the history of a critically ill patient. We cautiously suggest that the apparent "disorganization" could be reflecting a more sophisticated approach to handovers: a logical, efficient communication of complex information. Further research is required to examine whether alternative approaches to handover communication can be identified among the studied physicians and to identify features associated with good handovers. If such approaches and features can be identified, the implications for medical schools, training programs and practicing physicians would be substantial.

This study has several important strengths. First, to our knowledge, this is the only systematic description of medical handovers as performed by full-time faculty attending physicians. Experience is expected to engender wisdom and expertise, which, in turn, would contribute to the quality of handovers. Better understanding of high quality handovers will inform educational and quality improvement efforts in the area. Second, our description is one of very few (e.g. [7,43]) based on video or audio recordings of naturally occurring handovers (i.e., handovers that would have occurred whether or not the recorder was present).

Our study has four known limitations. First, participating physicians were aware of being recorded and this, similar to the Hawthorne Effect, might have influenced their behaviour. In addition, the set of sessions captured in the archive, while quite extensive, was nonetheless a convenience sample and thus not all handovers were captured during the study period. Furthermore, two of the participating physicians were also investigators in the study. These factors may have detracted from the internal validity of our findings. However, the studied handovers were recorded with the initial objective to create a data bank of naturally occurring handovers. Specific research questions did not exist and were not discussed prior to the completion of the data bank. It is likely that the recorded physicians attempted to do their best at the handovers; however, in the absence of any predefined outcome measures, participants could not have been biased in any way with respect to our research question. A second limitation is that the study was performed in a single centre and included a relatively small number of physicians. Our findings may, therefore, have limited generalizability to other ICUs. However, there is no institutional protocol around the format of handovers at Kingston General Hospital and all ten critical care physicians we studied have worked in other hospitals, which suggests that their behavior is likely more broadly representative. Third, there was substantial disagreement between coders regarding some elements of the standardized communication schemes. Although this may reflect the complexity of the handed over information and the inherent ambiguity of the proposed standard categories, it is nonetheless clear that standardized schemes were not used by the studied physicians according to any of the coders. All coders agreed that blocks of each content type were widely scattered throughout each patient discussion. We should perhaps not be surprised that coders have some difficulty agreeing as medical interns being taught such schemes also have difficulty following them [11]. In addition, there are some noticeable discrepancies among the coding according to the different communication standards. For example, whereas SBAR's *Recommendation *was absent in 55% of the handovers, SOAP's *Plan*, a seemingly equivalent construct, was absent in only 7.5%. Similarly, the combined proportion of SBAR's *Assessment *and *Recommendation *(21.9%) is much smaller, according to both coders, than the apparently similar content represented by SOAP's *Assessment *and *Plan *(37.6%). These can be explained by differences in the definitions of similar elements of the different standards (Figure 1). Finally, the quality of the recorded handovers was not assessed and consequently a relationship between handover quality and clinical outcomes could not be examined. Since a validated measurement tool for handover quality does not exist, further research is required to identify quality indicators for handover communication.

## Conclusion

Faculty-level critical care physicians did not follow commonly recommended medical communication schemes (SBAR, SOAP, MAN) during naturally occurring, videotaped handovers. Statements corresponding to the various mnemonic elements were scattered throughout the handovers without block structure or fixed order of arrangement and were sometimes entirely absent. Evidently, these widely promoted schemes do not fit the clinical work of handover within the present setting. Further research is required to examine whether a typical communication approach can be identified in the recorded handovers; to explore the role of questions and active involvement of incoming physicians; to evaluate the handovers' quality and relevance to meaningful clinical outcomes; and to identify features of high-quality handover communication.

## List of abbreviations

A+P: assessment and plan; CC: chief complaint; DOC: directives of care; ICU: intensive care unit; ID: identification; HPI: history of present illness; MAN: medical admission note; MHA: medications: habits and allergies; PE: physical examination; PMH: past medical history; SBAR: situation: background: assessment: recommendations; SD: standard deviation; SOAP: subjective, objective, assessment, plan.

## Competing interests

The authors declare that they have no competing interests.

## Authors' contributions

RI, CLB, MC, DH and MC conceived the study, participated in data analysis and interpretation and drafted the manuscript. RI also supervised data collection. AD helped with statistical analysis and interpretation.

All authors read and approved the final manuscript.

## Pre-publication history

The pre-publication history for this paper can be accessed here:

http://www.biomedcentral.com/1472-6963/12/11/prepub
